# Novel *TP53RK* variants cause varied clinical features of Galloway–Mowat syndrome without nephrotic syndrome in three unrelated Chinese patients

**DOI:** 10.3389/fnmol.2023.1116949

**Published:** 2023-02-16

**Authors:** Jing Chen, Gao-Bo Ye, Jin-Rong Huang, Min Peng, Wei-Yue Gu, Pin Xiong, Hong-min Zhu

**Affiliations:** ^1^Pediatric Rehabilitation Medicine, Wuhan Children's Hospital, Tongji Medical College, Huazhong University of Science and Technology, Wuhan, China; ^2^Department of Pediatrics, The Second Affiliated Hospital of Xi’an Jiaotong University, Xian, China; ^3^Ganzhou Women and Children Health Hospital, Ganzhou, China; ^4^Chigene Beijing Translational Medical Research Center Co., Ltd., Beijing, China

**Keywords:** *TP53RK* gene, Galloway–Mowat syndrome, nephrotic syndrome, proteinuria, global developmental delays

## Abstract

**Objectives:**

Galloway–Mowat syndrome-4 (GAMOS4) is a very rare renal-neurological disease caused by *TP53RK* gene mutations. GAMOS4 is characterized by early-onset nephrotic syndrome, microcephaly, and brain anomalies. To date, only nine GAMOS4 cases with detailed clinical data (caused by eight deleterious variants in *TP53RK*) have been reported. This study aimed to examine the clinical and genetic characteristics of three unrelated GAMOS4 patients with *TP53RK* gene compound heterozygous mutations.

**Methods:**

Whole-exome sequencing (WES) was used to identify four novel *TP53RK* variants in three unrelated Chinese children. Clinical characteristics such as biochemical parameters and image findings of patients were also evaluated. Furthermore, four studies of GAMOS4 patients with *TP53RK* variants were reviewed. In addition, clinical and genetic features were described after a retrospective analysis of clinical symptoms, laboratory data, and genetic test results.

**Results:**

The three patients showed facial abnormalities, developmental delays, microcephaly, and aberrant cerebral imaging. Furthermore, patient 1 had slight proteinuria, while patient 2 had epilepsy. However, none of the individuals had nephrotic syndrome, and all were alive for more than 3 years of age. This is the first study to assess four variants in the *TP53RK* gene (NM_033550.4: c.15_16dup/p.A6Efs*29, c.745A > G/p.R249G, c.185G > A/p.R62H, and c.335A > G/p.Y112C).

**Conclusion:**

The clinical characteristics of the three children with *TP53RK* mutations are significantly different from the known GAMOS4 traits, including early nephrotic syndrome and mortality mainly occurring in the first year of life. This study provides insights into the pathogenic *TP53RK* gene mutation spectrum and clinical phenotypes of GAMOS4.

## 1. Introduction

Galloway–Mowat syndrome (GAMOS) is a very rare recessively inherited disorder characterized by steroid-resistant nephrotic syndrome (SRNS) or early-onset NS, microcephaly, impaired brain development, and other features (Galloway and Mowat, 1968). GAMOS has high genetic heterogeneity. GAMOS (OMIM, PS251300) is associated with 10 different genes, including *WDR73*, *LAGE3*, *OSGEP*, *TP53RK*, *TPRKB*, *WDR4*, *NUP107*, *NUP133*, *GON7*, and *YRDC*. These genes are located in autosomes, except for the *LAGE3* gene with X-linked inheritance (Rosti et al., 2017; Braun et al., 2018; Fujita et al., 2018; Arrondel et al., 2019; Domingo-Gallego et al., 2019). A recent study showed that *PRDM15* gene variants can cause GAMOS (Mann et al., 2021). Galloway–Mowat syndrome-4 (OMIM, #617730) is a type of GAMOS caused by the TP53-regulating kinase (*TP53RK*) homozygous or compound heterozygous variant. However, very few GAMOS4 cases have been reported.

The *TP53RK* gene (OMIM, *608679) is located on chromosome 20q13.12 and has two exons. *TP53RK* encodes a 253-amino acid p53-related protein kinase or tp53 reactive kinase (PRPK or TPRK), belonging to the highly conserved kinase, endopeptidase, and other proteins of small size (KEOPS) complex (Treimer et al., 2022). PRPK promotes ATPase activity of the KEOPS complex, thus regulating N-6-threonyl-carbamoyl adenosine (t6A) synthesis and improving translational efficiency and accuracy (Treimer et al., 2022). PRPK is a human protein kinase involved in protein phosphorylation *via* protein serine/threonine kinase activity, p53 binding activity, transferase activity, and transferring phosphorus-containing groups. PRPK may play an essential role in cell apoptosis and cell cycle through phosphorylation of p53 serine-15 residue, thus enhancing transcriptional activity (Abe et al., 2001, 2006). Only nine GAMOS4 cases caused by eight deleterious variants in the *TP53RK* gene have been reported in four articles. Braun et al. (2017) discovered pathogenic *TP53RK* mutations (homozygous c.125G > A/p.G42D; homozygous c.728G > T/p.R243L; compound heterozygous c.179del/p.K60Sfs*61 and c.242C > G/p.T81R) in four patients from three unrelated GAMOS4 families with nephrotic syndrome onset before 1 year of age. A Korean case study also identified a homozygous mutation (c.194A > T/p. K65M) in the *TP53RK* of a GAMOS4 family, where all affected members showed very early onset nephrotic syndrome, microcephaly, facial abnormalities, and premature death (Hyun et al., 2018). A Chinese article reported a case of a 1-year-old girl with microcephaly, growth and intelligence retardation, and nephrotic syndrome. The whole-exome sequencing (WES) found that the child had compound heterozygous mutations c.107 T > C/p.L36P and c.728G > T/p.R243 (Zhuo et al., 2020). These studies show the infantile-onset nephrotic syndrome and serious manifestations of GAMOS4 with *TP53RK* deficiency. However, Treimer et al. (2022) described a 3-year-old boy with a homozygous *TP53RK* missense variant (c.163C > G/p.R55G), who presented with neurodevelopmental deficits and progressive microcephaly but no clinical history of renal failure.

In this study, four novel *TP53RK* pathogenic variants were identified in three Chinese children with GAMOS4 *via* WES. The children had heterozygous c.15_16dup/p. p.A6Efs*29, which was in compound heterozygous state with c.745A > G/p.R249G, c.185G > A/p.R62H, and c.335A > G/p.Y112C. One patient was alive up to 3 years of age, and another 6-year and the 3-month-old patient did not have nephrotic syndrome at the time of the most recent follow-up. This study provides different clinical characteristics associated with GAMOS4, thus improving the understanding of the *TP53RK*-associated phenotype.

## 2. Materials and methods

### 2.1. Ethical statement

The patients’ families provided informed consent for access to clinical data and genetic analysis, and this study was approved by the Ethics Committee of Wuhan Children’s Hospital (Grant No. 2022R043-E01).

### 2.2. Subjects

Three Chinese patients from unrelated families were enrolled in the study. Developmental delay (DD) was then evaluated between the ages of 5 months and 3 years. The patients were considered to have hereditary causes, and thus, blood samples from the families were collected for gene testing. The medical records of these patients, including clinical symptoms, physical examinations, laboratory tests, brain magnetic resonance imaging (MRI), and other radiological results, such as cognitive function, developmental assessment, and medical history, were also collected and analyzed.

### 2.3. Molecular analysis

First, peripheral blood (2 mL) was collected from the probands and their parents. A QIAamp DNA Blood Maxi Kit (Qiagen, Hilden, Germany) was then used to extract genomic DNA. The WES process and bioinformatics analysis were performed using Chigene (Beijing Chigene Translational Medicine Research Center Co., Ltd., Beijing). WES was performed using xGen® Exome Research Panel v1.0 capture probes (IDT, Conliwell, United States) with high-throughput sequencing on an Illumina NovaSeq 6,000 system (San Diego, United States) following the standard short-read length (PE150) sequencing protocol with at least 99% coverage. Quality control of the raw data was achieved using FastP software after the sequencing data had been cleaned and validated *via* the Illumina process. Burrows-Wheeler Aligner (BWA) software was used to compare the DNA sequence to the Ensembl reference genome GRCh37/hg19. Genomic Analysis Toolkit (GATK) software was used to analyze paired-end reads for the detection of single-nucleotide variants (SNVs), insertions, and deletions of <50 bp (small Indels). The genetic variations matching the patients’ phenotype were manually chosen after all WES variant data were sorted *via* automated genetic disease analysis using the Chigene Cloud Platform.^1^ This platform contains 35 public databases, including dbSNP, 1,000 genomes project, gnomAD, ESP, ExAC, Chigene in-house MAFs database, UCSC, RefGene, Ensembl, Gencode, LOVD, SWISS, Clinvitae, ClinVar, HGMD, OMIM, and Human Phenotype Ontology (HPO).^2^ The impact of the missense variant was assessed using Provean, SIFT, Polypen2, MutationTaster, M-Cap, and REVEL software packages. The functional change of splice variants was assessed using MaxEntScan, dbscSNV, GTAG, and SpliceAI software packages. Variants in dbSNP, 1,000 Genomes Project, gnomAD, ESP, and our in-house database with minor allele frequency (MAF) <0.05 were selected for segregation analysis during data interpretation. The function and the correlation with the disease phenotype of the remaining deleterious variant were compared with the OMIM database and published literature (Zheng et al., 2018; Dai et al., 2019; Han et al., 2020; Zhang et al., 2020). The pathogenicity of candidate disease-causing SNVs/small InDels in probands was classified based on the American College of Medical Genetics and Genomics (ACMG) plus Sequence Variant Interpretation Working Group international guidelines (SVI WG).^3^

Sanger sequencing confirmed the identified *TP53RK* variants in the three patients and their parents. The c.15_16dup primers are 5′- ATTTCCGAAAGGCCGGACA-3′ and 5′- GGGAGGCGTAACCACTTACA-3′ (product size 590 bp). The c.745A > G primers are 5′- AGATCTCATCTCATACTTGTAGCA-3′ and 5′- AGTAGACCTCTATGTCCTGGAGAA-3′ (product size 256 bp). The c.185G > A primers are 5′- AGCTTCGGAGCGCAGGGGAGT-3′ and 5′- AGCTACTACGCCGGCCGATGG-3′ (product size 353 bp). The c.335A > G primers are 5′- CCAATTGTCTTGGCTAAGTTGGAG-3′ and 5′- TCAGTTGTACCATTTCCTGTAGCA-3′ (product size 298 bp). PCR amplification was conducted on a Hema 9,600 PCR Thermo Cycler (Zhuhai Hema Medical Instrument Co., Ltd.) using a KAPA2G Robust HotStart PCR Kit (KAPA Biosystems). The products were then sequenced using the BigDye Terminator v1.1 Cycle Sequencing Kit on an ABI3730 sequencer (Thermo Fisher Scientific, Waltham, United States). DNASTAR was used to analyze the DNA sequences.

TP53RK protein sequences of eight different species from the NCBI database were used to evaluate the variant evolutionary conservation *via* multi-species alignment on blastp.^4^ The outcomes were graphically processed using MSAViewer comparison. The three missense variants were mapped onto the predicted human PRPK three-dimensional (3D) structure (AF-Q96S44-F1) of all 253 residues (NP_291028.3). The human KEOPS complex structural model, including TPRKB, PRPK, OSGEP, LAGE3, and GON7, was constructed by combining human PRPK-TPRKB complex structure (PDB ID 6WQX), predicted human PRPK structure (PDB ID AF-Q96S44-F1), and human OSGEP–LAGE3–GON7 complex structure (PDB ID 6GWJ). Hydrogen bond networks of the identified missense variants were visualized to evaluate their effect in ChimeraX.

## 3. Results

### 3.1. Clinical features

**Figure 1:**
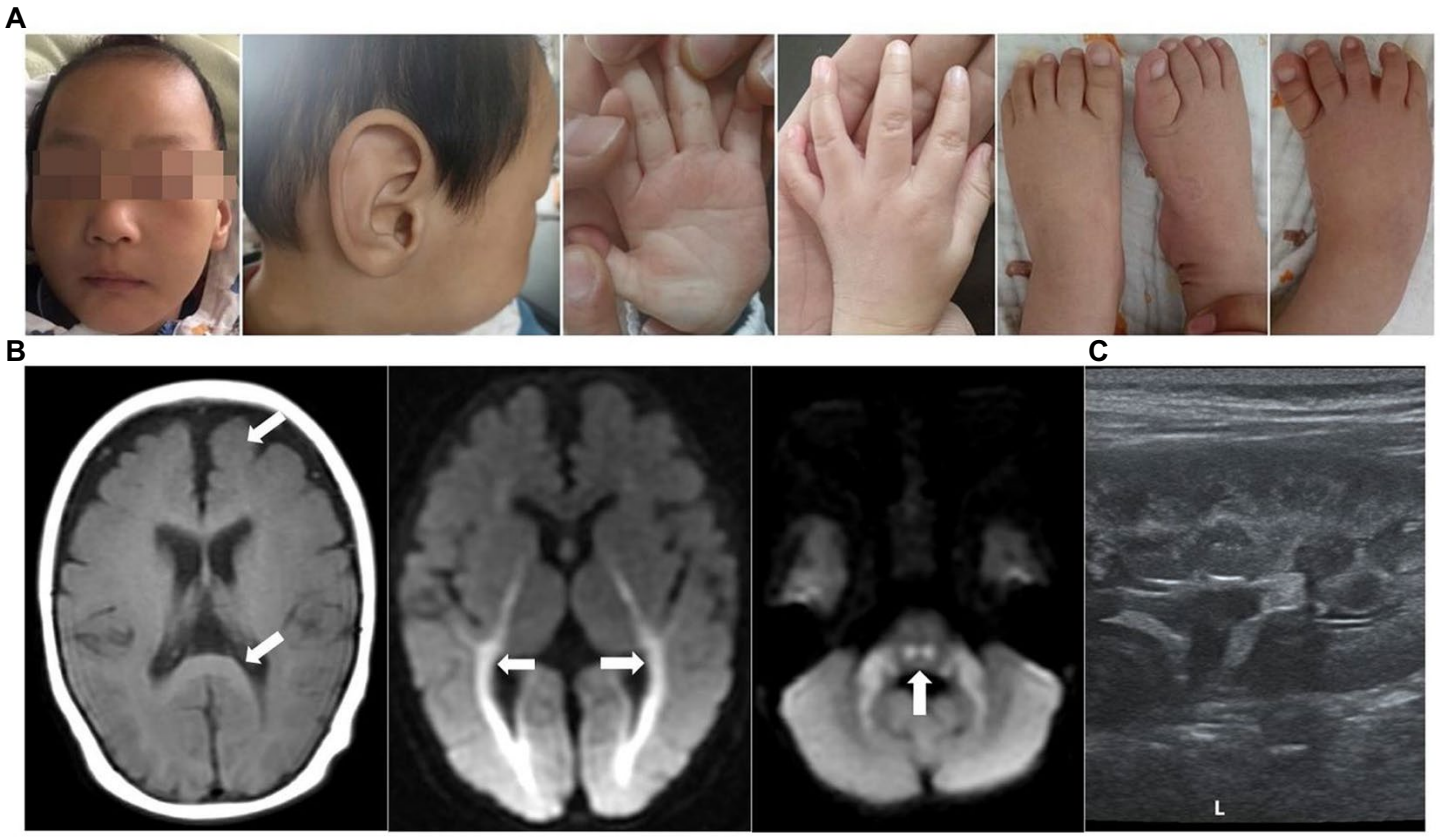
Clinical features of patient 1. **(A)** Images of faces, hands, and feet showed microcephaly, wide eye spacing, giant ear (large and soft external auricle), disordered palmar pattern, and conical fingers. **(B)** Brain MRIs performed at the age of 8 months: Axial T1-weighted and DWI images demonstrated an enlarged ventricular system, widened bilateral lateral fissure, anterior longitudinal fissure, and frontotemporal extracerebral space, as well as a slightly wider cerebral gyrus (white arrow). **(C)** Renal ultrasound disclosed enhanced renal cortex.

Patient 1 (P1) was an 8-month-old boy at the consultation, and he was the second child of a non-consanguineous couple. In addition, the couple had a first child, who was an 8-year-old girl with normal intellectual and motor development. The boy had intrauterine growth retardation and was delivered *via* full-term cesarean with a biparietal diameter significantly behind gestational age, a birth mass of 3,150 g, and a head circumference of 29 cm (<-3SD) at birth. The 8-month-old boy had facial abnormalities, with slight head deformity, a head circumference of 40.5 cm (<-3SD), wide eye spacing, a large external auricle, conical fingers, no skin edema, a dull pursuit of a vision, fleeting glances at objects in bright colors, was unresponsive to teasing, and poor sound source identification (Figure 1A). The boy could not turn over or sit alone, did not react to name-calling, could not roll over, and had high muscle tone in both upper limbs. The cranial MRI + DWI showed multiple intracranial abnormal signal shadows (bilateral cerebellar bridge arms, brainstem, bilateral lateral ventricles around the brain, the posterior limb of the internal capsule, and bilateral radiative crown areas were seen as stripes and small abnormal high signal shadows). Furthermore, the child had an enlarged ventricular system, widened bilateral lateral fissure, anterior longitudinal fissure, frontotemporal extra cerebral space, and a slightly widened brain gyrus (Figure 1B). A video EEG showed increased background slow activity, a few multifocal spikes, and sharp waves during sleep. Visual evoked potentials indicated extended P100 waves in both eyes. A fundus examination revealed no signs of optic nerve atrophy. Ultrasound of both kidneys showed echogenic enhancement of both cortices (Figure 1C). Bilateral ureteral ultrasound of the bladder, liver, and spleen did not reveal any abnormality. The child had normal left heart function tests and cardiac ultrasounds. The auditory brainstem response (ABR) and brainstem auditory evoked potential (BAEP) were used as standards. Proteinuria (2+), urine microprotein (1878.36 mg/L), β2 microglobulin (1.85 mg/L), urine calcium (Ca) (0.2 mmol/L), urine micro-to-creatinine ratio (370.69 mg/mmol), urine transferrin determination (43.6 mg/L), and serum triglycerides (2.71 mmol/L) were detected in urine. The developmental volume of Gesell Adaptive DQ = 28 was indicated at 8 months of age, indicating a serious global DD. The child received intensive rehabilitation intermittently for 3 months, after which he was discharged and was unsupervised. The child is now 3 years old with a height of 89 cm (<3rd), weight of 10.5 kg (<3rd), and head circumference of 42.5 cm (<−3SD). However, the child still has a severe motor, intellectual, and linguistic retardation, slightly puffed upper eyelids, hypotonia of the extremities, eating disorder, cannot sit or stand by himself, cannot respond to name-calling, cannot respond to teasing, and can only consume liquid and semisolid foods since he cannot chew.

**Figure 2:**
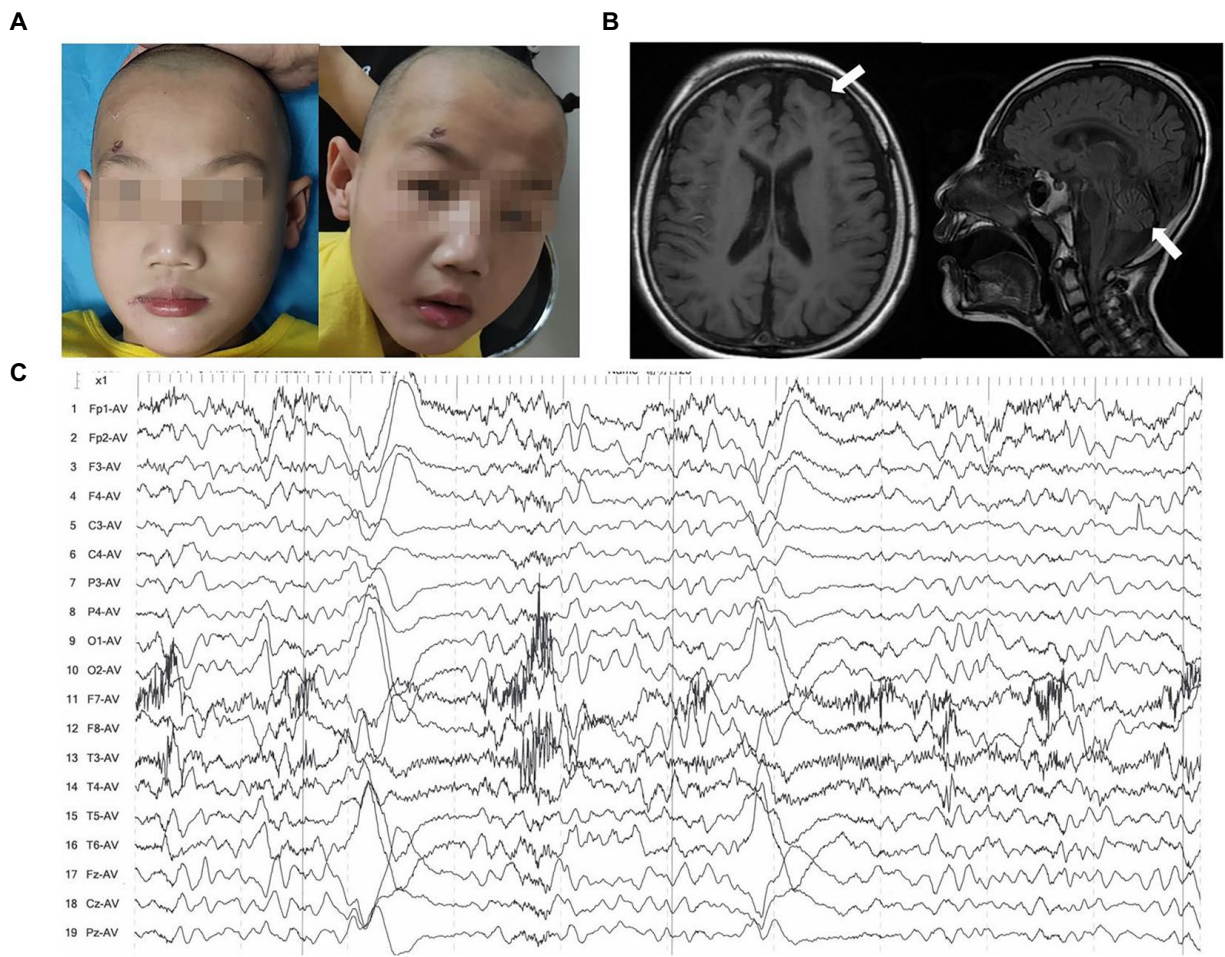
Clinical features of patient 2. **(A)** Images of faces showed microcephaly and wide eye spacing. **(B)** Brain MRIs performed at the age of 6 years: Axial T1-weighted and sagittal T2-flair images demonstrated enlargement and widening of the cerebral sulcus and cerebral fissure in the bilateral cerebral and cerebellar hemispheres (white arrow). **(C)** EEG indicated more spikes, slow spikes, multi-spikes, short-range paroxysms, and left–right asynchrony in the contralateral frontal pole and frontal and midline regions.

Patient 2 (P2) was a 3-year-old boy at the presentation. He was the firstborn delivered *via* full-term cesarean section with a birth mass of 3,000 g. The child had no history of asphyxia and hypoxia at birth and had an average head circumference at birth. The 3-year-old child had a facial abnormality, microcephaly, wide eye spacing, no skin edema, no hand or foot deformities, linguistic retardation, was unstable to sit alone, and had inconsistent muscular tone (Figure 2A). Cranial MRI showed a widening of extra cerebral space, macrocephaly, and microcephaly (Figure 2B). Seizures began when the patient was 2 years old and stopped at 1 year and 6 months after oral antiepileptic medication (oxcarbazepine, levetiracetam, and nitrazepam). A video EEG indicated an aberrant EEG, with more multi and slow spikes, short-range paroxysms, and left–right asynchrony in the contralateral frontal pole and frontal and midline regions during the waking and sleeping phases (Figure 2C). Longer, medium-high amplitude waves were interspersed with short-range paroxysms of sharp, spiky waves during the sleep period. Routine laboratory tests included blood lipid tests, liver and kidney function tests, and urine tests. The child underwent intermittent comprehensive rehabilitation for about 4 years. The child is currently 6 years and 2 months of age with a height of 120 cm (50th), weight of 20 kg (25th), and head circumference of 48 cm (−2SD). Nevertheless, the child has moderate to severe intellectual disability based on Wechsler Intelligence Scale for Children: VIQ = 52, PIQ = 45, FIQ = 41. After 4 years of intermittent comprehensive rehabilitation, he has poor posture, poor coordination, and ataxia movement, falls easily, cannot use active language or react to teasing, and can be amused without an eating disorder. The child can also sit alone for a short period and walk alone for a distance of about 20 m. Laboratory workup for urine routine at that age showed proteinuria was negative, renal function showed normal N-acetyl-β-D-glucosaminidase (7.3 U/L), urine microprotein (26.4 mg/L), urine transferrin determination (<2.33 mg/L), β2 microglobulin (<0.183 mg/L), serum albumin (40.2 g/L), and low blood creatinine (22.7 umol/L) and high alkaline phosphatase (229 IU/L).

Patient 3 (P3) was a 5-month-old girl at the consultation. She was the first delivery of the first pregnancy delivered *via* vaginal delivery after full-term (40^+4^ weeks) gestation and had no problems during pregnancy. She stayed 2 days in the NICU after birth due to “ductus arteriosus, newborn jaundice.” The head circumference at birth was 34 cm (<−3SD), and the body weight was 3,150 g. The 5-month-old girl had an abnormal facial appearance, with no hand and foot disfigurement, no skin edema, could not follow and hear, no midline movement of hands, had an unstable vertical head, a transient head elevation of 45 degrees in a prone position, and could not turn over. The cranial MRI + DWI imaging ruled out genetic coyote encephalopathy. Furthermore, multiple patchy and punctate T2, slightly high signals in bilateral radiating coronas, bilateral internal capsule hind limbs, bilateral paraventricular white matter, midbrain, protocerebrum, and dorsal protocerebrum were detected. The cerebellum was hypoplastic, and the bilateral frontotemporal subarachnoid gap was expanded. In addition, the bilateral inferior of the cerebellar hemispheres was reduced and myelin production was regressed (AIMS assessment: <5th). Laboratory tests showed that the urine routine, protein, liver, kidney, and lipid functions were normal. The patient received extensive intermittent rehabilitation for 1 month and stopped receiving any treatment after leaving.

### 3.2. Genetic findings

**Figure 3:**
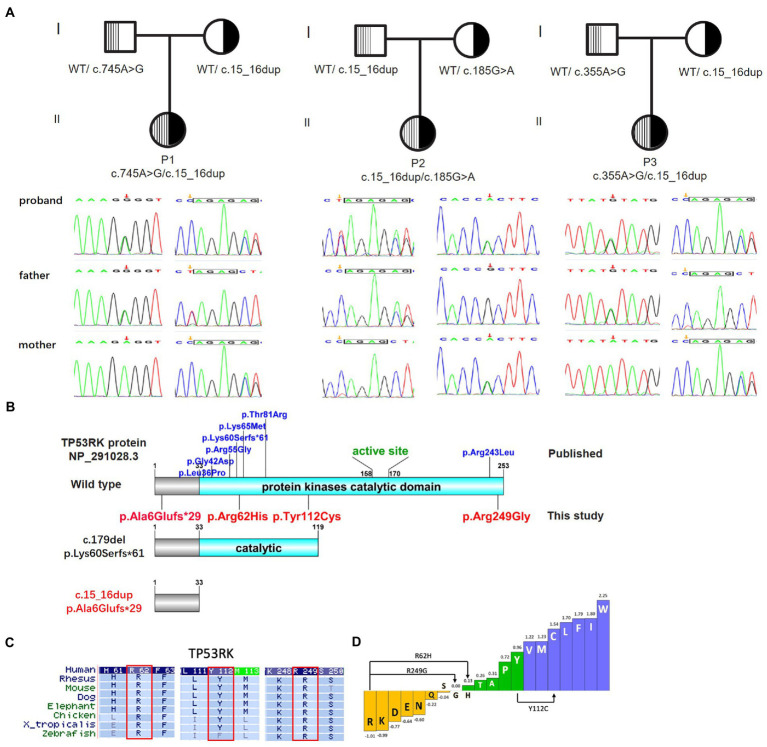
Biallelic variants in *TP53RK* cause Galloway–Mowat syndrome in three unrelated Chinese patients without the nephrotic syndrome. **(A)** The pedigree of the three unrelated GAMOS4 families for whole-exome sequencing (WES) and Sanger sequencing to verify the identified *TP53RK* variants. **(B)** Protein domain pattern map of human TP53RK and position of mutations. **(C)** Multiple amino acid sequence alignment of TP53RK5 among different species on missense variants (R62H, Y112C, and R249G). **(D)** Enhanced hydrophobicity properties of three missense variants (R62H, Y112C, and R249G).

Four novel variants were identified in *TP53RK* of the three families *via* WES (one frameshift variant and three missense variants). The probands had a frameshift variant NM_033550.4: c.15_16dup/p. A6Efs*29 (dbSNP: rs774069989). The missense variants c.745A > G/p.R249G, c.185G > A/p.R62H (dbSNP: rs750395029), and c.335A > G/p.Y112C (dbSNP: rs1292100340) were detected in Trans with c.15_16dup of P1, P2, and P3, respectively. No other pathogenic or likely pathogenic variants were detected in DD-or GAMOS-associated genes. The variants in the three families were further validated *via* sanger sequencing, and the result indicated that all children were autosomal recessive inherited from their parents who were heterozygous carriers for the variants in *TP53RK* (Figure 3A). The four variants have never been documented in the HGMD and the allele distribution frequency (MAF) is <0.0005 in the public population databases. The variant c.15_16dup truncates protein, thus destroying the kinase domain (Figure 3B). Provean, SIFT, Polyphen2_HDIV, and Polyphen2_HVAR predicted that the variants R249G, R62H, and Y112C in the *TP53RK* gene are deleterious to protein function. Moreover, the three missense variants are conserved across several species (Figure 3C) and can increase hydrophobicity (Figure 3D). The microcephaly and impaired brain development of the patients are similar to the associated manifestation of GAMOS4. Furthermore, c.15_16dup is classified as pathogenic (PVS1 + PM2 + PP4), while R249G, R62H, and Y112C are likely pathogenic (PM2 + PM3 + PP3 + PP4) according to the ACMG criteria of variants (Riggs et al., 2020).

**Figure 4:**
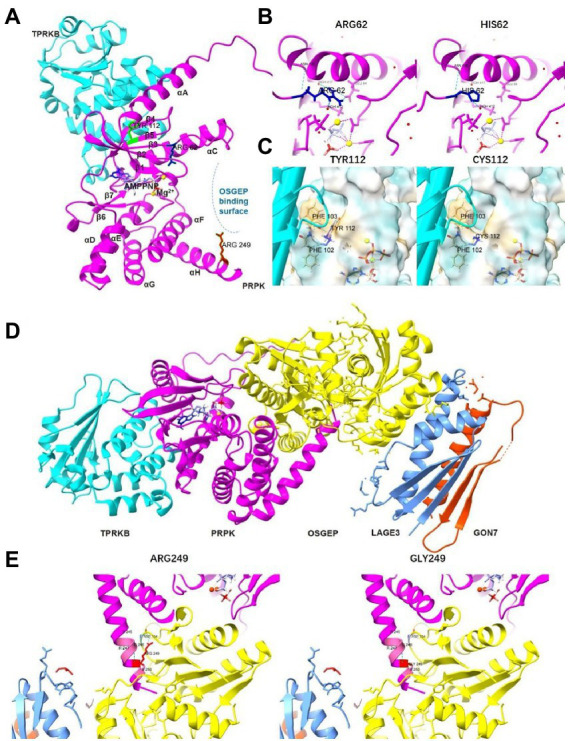
The effect of the three missense variants on the TP53RK protein. **(A)** The panoramic 3D map of wild-type TP53RK protein and position of mutations. **(B)** Prediction of the change in the 3D structure of PRPK by TP53RK R62H variant. **(C)** Prediction of the change in the 3D structure of PRPK-TPRKB complex by TP53RK Y112C variant. **(D)** The panoramic 3D map of a wild-type human KEOPS complex structural model, composed of TPRKB, PRPK, OSGEP, LAGE3, and GON7. **(E)** Prediction of the change in the 3D structure of KEOPS complex by TP53RK R249G variant.

p53-related protein kinase adopts a bilobate kinase domain with the N-lobe and the C-lobe based on the structure data (UniProtKB ID Q96S44). A cleft between the N-and C-lobes harbors the ATP analog, AMP-PNP. Arg62 is located within the β3-αC linker, close to the PRPK ATP binding pocket (Figure 4A). Analysis of 3D modeling revealed that the substitution of Arg62 to histidine can disrupt two hydrogen bonds that bind Arg62 with Glu84 of helix αC, which plays important catalytic roles (Figure 4B). These findings show that R62H can change the ATP binding pocket structure. Tyr112 is part of strand β4 and on the interface between PRPK and TPRKB (Figure 4A). Y112C wild-type Tyr112 and mutant Cys112 retain three hydrogen bonds unchanged with Phe102 and Phe103 of strand β5, which form hydrophobic interactions on the TPRKB helix. However, Y112 can change the π-π stacking interaction between the benzene ring of Tyr112 and Phe103 (Figure 4C), indicating that Y112C changes the amino acid side chain and may alter the normal spatial stability of the interaction between PRPK and TPRKB. Arg249 is located in the PRPK helix αH close to the OSGEP catalytic center based on the model of the human PRPK–TPRKB–OSGEP–LAGE3–GON7 complex (Figures 4A,D). Mutation of Arg249 to Gly can disrupt PRPK interaction with OSGEP as the hydrogen bond between PRPK Arg249 and OSGEP Asp154 disappears (Figure 4E). Therefore, the three probands were considered as GAMOS4 caused by novel compound heterozygous *TP53RK* variants.

## 4. Discussion

Galloway–Mowat syndrome is a genetic disease characterized by various developmental and physical abnormalities. GAMOS is caused by mutations in several genes reported in 86 families, including 60 cases (49.2%) of *WDR73* mutation, 26 cases (21.3%) of *OSGEP* mutation, 13 cases (10.6%) of *GON7* mutation, 8 cases (6.6%) of *TP53RK* modification, 5 cases (4%) of *NUP107* conversion, 3 cases (2.5%) of *YRDC* mutation, 3 cases (2.5%) of *LAGE3* mutation, 2 cases (1.6%) of *TPRKB* transformation, 1 case (0.8%) of *WDR4* mutation, and 1 case (0.8%) of *NUP133* mutation (Rosti et al., 2017; Fujita et al., 2018; Wang et al., 2018; Arrondel et al., 2019; Domingo-Gallego et al., 2019; El Younsi et al., 2019; Racine and Golden, 2021). GAMOS has very heterogeneous signs and symptoms, including microcephaly (small head size), impaired motor development, intellectual disability, seizures, nephrotic syndrome, hiatal hernia, optic atrophy, and other physical abnormalities.

Galloway–Mowat syndrome-4 caused by the *TP53RK* gene is mainly characterized by microcephaly, nephrotic syndrome, and severe central nervous system abnormalities (severe global developmental delay, cerebral atrophy, visual impairment, and dystonia). The clinical features and *TP53RK* variants in the three patients and previously published cases with documented GAMOS4 are summarized in Table 1. The first GAMOS4 study found four patients from three consanguine families of different origins (European, South African, Indian, Thailand, and Morocco; Abe et al., 2001). In that study, all symptoms appeared earlier, especially renal phenotype (diffuse mesangial sclerosis or focal-segmental glomerulosclerosis). Two patients, B77_21 and B77_22, were siblings who developed early proteinuria at 2 months and died at 11 and 2.5 months of age. The other two patients, N2984 and N2194, had proteinuria at the age of 1 year and 10 months and later died of end-stage renal disease (ESRD) at the age of 2.5 years and 3 years, respectively. The second study reported a Korean GAMOS non-consanguineous family with three affected siblings (II-1, II-2, and II-4) related to a homozygous *TP53RK* mutation genetic testing was not conducted in one case (II-1; Abe et al., 2006). Three of the four children in the family showed similar phenotypes, including early-onset nephrotic syndrome, microcephaly, facial deformity, and early death (age of death: 10 months, 21 days, and 25 days, respectively). Another Chinese article (2020) found a 1-year-old girl with hypoalbuminemia, hyperlipidemia, massive proteinuria, renal tubule damage, low level of immunoglobulin, special facies (broad and flat nose, too wide eye space), speech delay, continuous tremor of hands and feet, and could not sit alone (Braun et al., 2017). The patient was diagnosed with cerebral palsy and nephrotic syndrome before genetic testing. The above-reported individuals displayed early-onset nephrotic syndrome within the first year of life and died before the age of 3 from renal illness. In this study, the three patients had some typical features of this syndrome, such as small head circumferences (primary microcephaly), abnormalities in their cranial imaging (cerebral hypoplasia), and a DD (severe intellectual motor dysfunction and speech impairment). However, the three patients are now over 3 years of age, especially P2, who are over 6 years of age, demonstrating the high degree of heterogeneity in *TP53RK*-related GAMOS4. The three children also had strabismus and dystonia, but none had nephrotic syndrome. However, P1 had asymptomatic proteinuria. The latest documented Turkey case also had little renal involvement and survived until the age of 3 years and 7 months (Treimer et al., 2022). Moreover, cranial imaging of individuals was variable. Abe et al. (2001) found patients with cerebellar hypoplasia (B77_22), polymicrogyria (B77_21 and B77_22), cerebral atrophy (N2984), and bilateral myelination defects (N2194). Axial T2-weighted MRI of case II-2 showed a pattern of simplified sulcation (Abe et al., 2006). Brain MRIs of the Turkey case detected cortical atrophy with increased extra-axial spaces, thin corpus callosum, simplified gyral pattern, mild cerebellar atrophy, and diffuse T2-hypointense signal of the thalamus (Treimer et al., 2022). In this study, P2 and P3 exhibited cerebellar atrophy, while P1 did not show cerebellar hypoplasia but had numerous aberrant signal shadows in the brain and enlargement of the cerebral gyrus, a typical cranial lesion that has not been documented. Abe et al. (2001) showed that two cases had mixed seizures while only P2 had concomitant seizures in this study. P1 did not have epilepsy at the time of reporting but had an abnormal EEG, which does not rule out the occurrence of unmonitored seizures. EEG results of P3 were not detected. Furthermore, the three patients had abnormal facial features, primarily wide eye spacing, ear deformities, and small jaws. P1 also had hand and foot deformities, which appeared as conical fingers and foot entropion (the first phenotypic feature detected in GAMOS4 patients).

**Table 1:** Clinical characteristics and variants of *TP53RK*-associated GAMOS4.

Patients	B77-21	B77-22	N2984	N2194	II-1	II-2	II-4	Shi et al	Treimeretal	P1	P2	P3
Gender	Male	Female	Female	Female	Female	Male	Male	Female	Male	Male	Male	Female
Age at death	11 months	2.5 months	2.5 years	3 years	10 months	21 Days	25 days	ND	ND	Still alive	Still alive	ND
Ethnicity	Europe	Europe	Thailand	Morocco	Korea	Korea	Korea	China	Turkey	China	China	China
Consanguinity	Yes	Yes	Yes	Yes	No	No	No	No	Yes	No	No	No
Biopsy	FSGS (1y)	DMS	ND	Inadequate	FSG (2w)	NP	NP	NP	NP	NP	NP	NP
Renal phenotype	Congenital NS	Congenital NS	NS	NS	NS (8d)	NS (1d)	NS (1d)	NS	Proteinuria	Proteinuria	−	−
Microcephaly	+	+	+	+	+	+	+	+	+	+	+	+
Brain MRI	Polymicrogyria	Cerebellar hypoplasia, polymicrogyria	Cerebral atrophy	Bilateral myelination defects	Progressive brain atrophy	Lissencephaly, cerebellar hypoplasia, pontine hypoplasia	Lissencephaly	ND	Simplified gyral pattern, cerebellar atrophy	Multiple abnormal intracranial signal shadows	Cerebellar atrophy	Cerebellar hypoplasia
GDD	+	+	+	+	ND	ND	ND	+	+	+	+	+
Other brain phenotype	Seizures, hypotonia, spasticity	Seizures, hypotonia	−	Hypotonia	−	−	−	Hypotonia	Hypotonia, truncal and peripheral spasticity	Hypotonia, abnormal EEG	Seizures, ataxia	Hypotonia
Facial features	Plagiocephaly, prominent glabella, large ears	Dysmorphism	Large and protruding ears, hypertelorism	ND	Low-set ears, hypertelorism	Narrow forehead, almond eyes, depressed nasal bridge, hypertelorism, high-arched cleft palate, large low-set ears, micrognathia	Low-set ears, hypertelorism	Flat nasal bridge, wide eye spacing	Narrow forehead, deep-set eyes, bilateral esotropia, prominent nasal bridge, large ears	large low-set ears, wide eye spacing, internal strabismus, prominent forehead, low nasal bridge	Wide eye spacing, drooping outer corners of both eyes, prominent forehead, low nasal bridge	Narrow forehead
Other features	Short stature, tapered fingers, feeding difficulty, multiple hypo and hyper-pigmented macules over the abdomen and limbs	−	−	Reduced VEP	Hiatal hernia, gastric volvulus	Arachnodactyly, clasped thumb	Intrauterine growth retardation	Tremor of hand and foot	Arachnodactyly, clinodactyly, feeding difficulty, gastroesophageal reflux, cryptorchidism, hypoplastic scrotum	Intrauterine growth retardation, gastroesophageal reflux, feeding difficulty, prolonged VEP latency	−	−
Nucleotide change (zygosity)	c.179del (het, m) c.242C > G (het, p)	c.179del (het, m) c.242C > G (het, p)	c.125G > A (hom)	c.728G > T (hom)	ND	c.194A > T (hom)	c.194A > T (hom)	c.107 T > C (het, m) c.728G > T (het, p)	c.163C > G (hom)	c.15_16dup (het, m) c.745A > G (het, p)	c.185G > A (het, m) c.15_16dup (het, p)	c.15_16dup (het, m) c.335A > G (het, p)
Amino acid change	p.K60Sfs* p.T81R	p.K60Sfs* p.T81R	p.G42D	p.R243L	ND	p.K65M	p.K65M	p.L36P p.R243L	p.R55G	p.A6Efs*29 p.R249G	p.R62H p.A6Efs*29	p.A6Efs*29 p.Y112C

ND, no data; NP, not performed; NS, nephrotic syndrome; ESRD, end-stage renal disease; FSGS, focal segmental glomerulosclerosis; DMS, diffuse mesenteric sclerosis; MRI, magnetic resonance imaging; GDD, global developmental delay; VEP, visual evoked potential; het m, maternal heterozygous; het p, paternal heterozygous; hom, homozygous.

The TP53-regulating kinase (PRPK and TP53RK) is a component of the KEOPS complex essential for tRNA (t6A) modification, transcription control, and telomere regulation (Treimer et al., 2022). KEOPS complex comprises five subunits: LAGE3, OSGEP, PRPK, TPRKB, and the recently discovered C14orf142/*GON7* (Wan et al., 2017). LAGE3, OSGEP, and PRPK are found in human podocytes and may be the minimum set required for the transfer of the threonyl-carbamoyl group to the adenosine at position 37 (t_6_A37) in tRNAs that recognize ANN codons. TPRKB (TP53RK-binding protein) significantly increases the activity between its interactions with PRPK N-lobe. PRPK-TPRKB has autophosphorylation activity, which is switched to ATPase activity after the addition of OSGEP. The PRPK C-terminus of helix αH can play a supporting role in the OSGEP catalytic center (Daugeron et al., 2011). Most typical GAMOS patients have a defect in the KEOPS complex. Knockdown of OSGEP, TP53RK, and TPRKB can inhibit cell proliferation and reduce cell migration of human podocytes, impair translation, upregulate endoplasmic reticulum (ER) stress, activate DNA damage response (DDR) signaling, increase apoptosis, and decrease actin cytoskeleton network (Braun et al., 2017). Therefore, mutations in the KEOPS complex significantly affect the etiology of GAMOS. Although Ensembl describes two transcript variants (ENST00000372114.3 and ENST00000372102.3) encoding two potential isoforms (ENST00000372114.3-253aa and ENSP00000361174-121aa) of *TP53RK*, the clinical pathological significance of each transcript isoform of *TP53RK* in GAMOS is unknown. In this study, the variants were described using the NM_033550 transcript reference sequence based on ACMG guidelines and reported literature.

To date, only 12 *TP53RK* pathogenic variants have been identified in eight GAMOS families with detailed clinical data. Nine missense and two frameshift mutations in *TP53RK* have been found in 12 individuals (Table 1). All mutations occur in the catalytic domain of protein kinases (residues33-253), except for the novel frameshift c.15_16dup, which occurs at the N-terminus (Figure 3B). In this study, the three children had the *TP53RK* frameshift c.15_16dup. The allele distribution frequency (MAF) of this variant is 0.002% (EXAC, GnomAD), 0.009% (EXAC Asia), 0.011% (GnomAD Asia), and 0.0405% (China’s in-house local database), indicating that this variant is common in East Asian or Chinese populations. Moreover, the c.15_16dup allele in the three children was absent in other populations, strongly suggesting that it may be a founder mutation at a low carrier frequency. Although this variant is very rare in the general population, it has been detected previously for dbSNP identifier rs774069989, most likely of South Korean ancestry. It is also associated with microcephaly. In addition, this variant was not present in the available data from the 1,000 Genomes Project but was present in the heterozygous state in 54 individuals from China’s in-house local database (see text footnote 1). Therefore, screening of homogeneous individuals and variant-specific core haplotypes should be conducted to further investigate the founder effect hypothesis. Another reported frameshift c.179del was found in a compound heterozygous state with T81R in 2 sibs, which inhibits TP53RK interaction with TPRKB according to immunoprecipitation tests (Braun et al., 2017). c.179del can produce a 113-amino acid protein that can partially destroy the kinase domain, while c.15_16dup can form a 33-truncated protein without the kinase domain. Therefore, c.179del may cause the similarity in the three patients, including facial dysmorphism, microcephaly, DDs, brain anomalies, a propensity for seizures, and no early-onset nephrotic syndrome. These missense variants are located in a highly conserved protein kinase domain and can attack the disease. Although high-resolution structures of the human KEOPS complex are unavailable, human PRPK, X-ray diffraction structure of human PRPK-TPRKB complex, and human OSGEP/LAGE3/GON7 complex (predicted by modeling based on AlphaFold) revealed that R249G, R62H, and Y112C can weaken interactions and destabilize the KEOPS complex by impeding electrostatic interactions (Figure 4E). Nevertheless, further research is needed to fully understand the functional structural domain of TP53RK protein and identify additional genotype–phenotype associations since past medical history, and the progression of clinical symptoms is unclear in some patients. The discovery of the four variants broadens the range of pathogenic mutations in GAMOS4.

The severity of the renal symptoms of GAMOS ranges from mild non-nephrotic proteinuria to SRNS, quickly followed by end-stage renal disease and death. Renal disease is the only known cause of death among GAMOS patients. The impact of the *WDR73* variant on GAMOS1 mortality is about 36.7% (22/60; Colin et al., 2014; Ben-Omran et al., 2015; Jinks et al., 2015; Vodopiutz et al., 2015; Rosti et al., 2016). Furthermore, there is no cure for this kidney disease, and all treatments are only supportive. The age at death range from 1.4 to 28 years (mean, 8.4 years). Herein, P1 only had minor chronic proteinuria with no nephrotic syndrome signs, while P2 and P3 did not show any kidney disease. In contrast, seven of the previously reported GAMOS4 patients had early onset nephrotic syndrome and passed away from renal insufficiency before the age of 3 years (mean, 1.1 years). The prognosis of GAMOS patients may be correlated with the early onset of the nephrotic syndrome. The patient’s intellectual and motor development is relatively mild if the onset of the syndrome is late, and their renal function also gradually declines. However, the patient can live longer if hormonal or immunosuppressive therapy is effective (Udler et al., 1997). To date, the oldest GAMOS survivor without nephropathy was about 35 years. The survivors had GAMOS1 caused by the *WDR73* mutation (Jiang et al., 2017). To the best of our knowledge, P2 is the oldest child that has ever survived GAMOS4 caused by the *TP53RK* gene variant. The three patients did not have nephrotic syndrome, and only P1 had slight proteinuria, indicating that their overall survival was relatively long. However, urine cycle, liver and kidney function, and a renal ultrasound should be dynamically monitored. In addition, the detection of a combination of recurrent urinary tract infections and renal tissue biopsy is necessary.

In conclusion, four novel *TP53RK* variants were identified in three unrelated neurodevelopmental disorder patients using WES. This study provides additional clinical findings of GAMOS4 and enriches the mutation spectrum of the *TP53RK* gene. Nevertheless, this study provides a basis for the future clinical diagnosis and identification of GAMOS4 disease.

## Data availability statement

The original contributions presented in the study are publicly available. Information about TP53RK variants in this study can be accessed online through the lovd database using id #0000918160 (c.15_16dup) (https://databases.lovd.nl/shared/variants/0000918160#00021665), #0000918161(c.745A>G) (https://databases.lovd.nl/shared/variants/0000918161#00021665), #0000918162(c.185G>A) (https://databases.lovd.nl/shared/variants/0000918162#00021665), #0000918165(c.335A>G) (https://databases.lovd.nl/shared/variants/0000918165#00021665).

## Ethics statement

The studies involving human participants were reviewed and approved by Wuhan Children’s Hospital, Tongji Medical College, Huazhong University of Science and Technology, Wuhan, China. Written informed consent to participate in this study was provided by the participants’ legal guardian/next of kin. Written informed consent was obtained from the minor(s)' legal guardian/next of kin for the publication of any potentially identifiable images or data included in this article.

## Author contributions

JC and H-mZ designed the study. PX, G-BY, and J-RH gathered clinical information from the family members and drafted the manuscript. MP and W-YG performed the sequencing, as well as analyzed and interpreted the data. All authors coordinated the study coordination, revised the manuscript, and read and approved the final version of the manuscript.

## Funding

This research was supported by the Hubei Provincial Science and Technology Plan Project for the Clinical Research Center of Neurodevelopmental Disorders in Children (No. 2022DCC020).

## Conflict of interest

MP and W-YG were employed by the Beijing Chigene Translational Medical Research Centre Co. Ltd.

The remaining authors declare that the research was conducted in the absence of any commercial or financial relationships that could be construed as a potential conflict of interest.

## Publisher’s note

All claims expressed in this article are solely those of the authors and do not necessarily represent those of their affiliated organizations, or those of the publisher, the editors and the reviewers. Any product that may be evaluated in this article, or claim that may be made by its manufacturer, is not guaranteed or endorsed by the publisher.

## Footnotes

^1^ https://cloud.chigene.org:18081/#/login

^2^ https://hpo.jax.org/

^3^ https://www.clinicalgenome.org/working-groups/sequence-variant-interpretation/

^4^ https://blast.ncbi.nlm.nih.gov/Blast.cgi?PAGE=Proteins&PROGRAM=blastp&PAGE_TYPE=BlastSearch&BLAST_SPEC=
